# Nrf2-Inducers Counteract Neurodegeneration in Frataxin-Silenced Motor Neurons: Disclosing New Therapeutic Targets for Friedreich’s Ataxia

**DOI:** 10.3390/ijms18102173

**Published:** 2017-10-18

**Authors:** Sara Petrillo, Emanuela Piermarini, Anna Pastore, Gessica Vasco, Tommaso Schirinzi, Rosalba Carrozzo, Enrico Bertini, Fiorella Piemonte

**Affiliations:** 1 Unit of Neuromuscular and Neurodegenerative Diseases, IRCCS Bambino Gesù Children’s Hospital, Viale San Paolo 15, 00146 Rome, Italy; sara.petrillo@opbg.net (S.P.); emanuela.piermarini@opbg.net (E.P.); rosalba.carrozzo@opbg.net (R.C.); enricosilvio.bertini@opbg.net (E.B.); 2 Drexel University College of Medicine, 2900 Queen Lane, Philadelphia, PA 19129, USA; 3 Laboratory of Biochemistry, IRCCS Bambino Gesù Children’s Hospital, Viale San Paolo 15, 00146 Rome, Italy; anna.pastore@opbg.net; 4 Movement Analysis and Robotics Laboratory (MARLab), Neurorehabilitation Unit, Department of Neurosciences, IRCCS Bambino Gesù Children’s Hospital, Via Torre di Palidoro, Passoscuro Fiumicino, 00050 Rome, Italy; gessica.vasco@opbg.net (G.V.); tommaso.schirinzi@opbg.net (T.S.)

**Keywords:** oxidative stress, Nrf2, Friedreich’s Ataxia, sulforaphane, dimethyl fumarate, inducers

## Abstract

Oxidative stress is actively involved in Friedreich’s Ataxia (FA), thus pharmacological targeting of the antioxidant machinery may have therapeutic value. Here, we analyzed the relevance of the antioxidant phase II response mediated by the transcription factor Nrf2 on frataxin-deficient cultured motor neurons and on fibroblasts of patients. The in vitro treatment of the potent Nrf2 activator sulforaphane increased Nrf2 protein levels and led to the upregulation of phase II antioxidant enzymes. The neuroprotective effects were accompanied by an increase in neurites’ number and extension. Sulforaphane (SFN) is a natural compound of many diets and is now being used in clinical trials for other pathologies. Our results provide morphological and biochemical evidence to endorse a neuroprotective strategy that may have therapeutic relevance for FA. The findings of this work reinforce the crucial importance of Nrf2 in FA and provide a rationale for using Nrf2-inducers as pharmacological agents.

## 1. Introduction

Oxidative stress and mitochondrial dysfunction play a critical role in the pathogenesis of Friedreich’s Ataxia (FA), an autosomal-recessive neurodegenerative disease due to homozygous GAA expansions in the first intron of the frataxin gene on chromosome 9. Following epigenetic alterations, the expression of the mitochondrial protein frataxin is consistently decreased in patients leading to iron overload, impairment of iron–sulfur cluster biosynthesis and generation of reactive oxygen species. Neurodegeneration usually starts in dorsal root ganglia, where reduction of large neurons and loss of myelinated fibers are evident. The axonopathy arises from a retrograde dying-back mechanism due to defective axonal and mitochondrial transport along the axon [1,2,3]. In this study, we determined whether NF-E2-related factor-2 (Nrf2), master regulator of redox homeostasis, might be a therapeutic target for the disease.

Nrf2 modulates the expression of many genes involved in the antioxidant defense by the activation of the enzymes constituting the phase II response [4]. These genes code heme oxygenase-1 (HO-1), NAD(P)H quinone oxidoreductase 1 (NQO1), Cu/Zn and Mn-superoxide dismutases (SOD 1,2) and the γ-glutamyl cysteine ligase (GCL-C and GCL-M), among several others [5,6,7,8]. Under physiological conditions, Nrf2 is bound to the protein Keap1 in the cytosol, where it undergoes degradation by means of the ubiquitin–proteasome pathway. Numerous drugs form adducts with specific cysteines on Keap1, modifying the Nrf2/Keap1 complex and inducing the Nrf2 stabilization, nuclear translocation and up-modulation of phase II gene transcription [7]. Variations in the intracellular redox state can, therefore, transiently modify the activity of Nrf2 and its activation has been shown to counteract a number of pathological mechanisms associated with several neurodegenerative diseases, such as Alzheimer’s disease, Parkinson’s disease (PD), Amyotrophic Lateral Sclerosis, Huntington’s disease and Multiple Sclerosis [9].

Several important findings connect Nrf2 with FA. Nrf2 signaling is defective in both FA patients and in preclinical models of frataxin deficiency, leading to increased sensitivity to oxidative stress [2,10,11]. Frataxin-dependent Nrf2 defects have been reported in the DRG and cerebella of YG8R hemizygous mice [2] and in multiple cell models, including DRG, Schwann and HeLa cells [2,10]. Impaired nuclear Nrf2 translocation, associated with a decreased expression of its antioxidant target genes, including SOD1 and SOD2, glutathione transferases, HO-1 and GCL-M, has been further described in FA cultured fibroblasts and in neuroblastoma-derived cell lines (SKNAS) [10,11]. Nrf2 is also known to positively regulate Nrf1, a marker of mitochondrial biogenesis, thus its induction may additionally contribute to activate mitochondrial functions in FA [12].

In the present study, we chose sulforaphane (SFN) to target Nrf2 in a frataxin-silenced model of cultured motor neurons generated and characterized in our laboratory [13,14]. SFN is an isothiocyanate found in cruciferous vegetables that has gained attention as a chemopreventive and anti-inflammatory compound [15,16,17]. Jazwa et al. [18] showed that SFN crosses the blood–brain barrier when intra-peritoneal injected in the MPTP mouse model of PD, and it was detected in the brain already 15 min after injection. SFN induced Nrf2-dependent phase II response in the basal ganglia and protected against nigral dopaminergic cell death, astrogliosis, and microgliosis. In addition, SFN has been shown to ameliorate motor function deficits in a rotenone-induced mouse model of PD [19].

To confirm and compare the effect of Nrf2-inducers on FA neuroprotection, we also treated frataxin-silenced neurons with dimethyl fumarate (DMF), a drug already in use for the treatment of multiple sclerosis and recently proposed as a therapy for PD [20]. As with SFN, DMF crosses the blood–brain barrier and activates Nrf2 in the brain, evoking neuroprotective effects in a PD mouse model.

Our findings indicate that both Nrf2-inducers significantly up-regulate Nrf2 and its target genes in frataxin-deficient motor neurons. This activation contributes to stabilizing the redox environment in FA by targeting the repletion of reduced glutathione (GSH) and mitigating reactive oxygen species (ROS)-related damage, thus ultimately counteracting the neurodegeneration.

## 2. Results

### 2.1. SFN Upregulates Nrf2 in Frataxin-Silenced Motor Neurons (shFxn)

**Figure 1:**
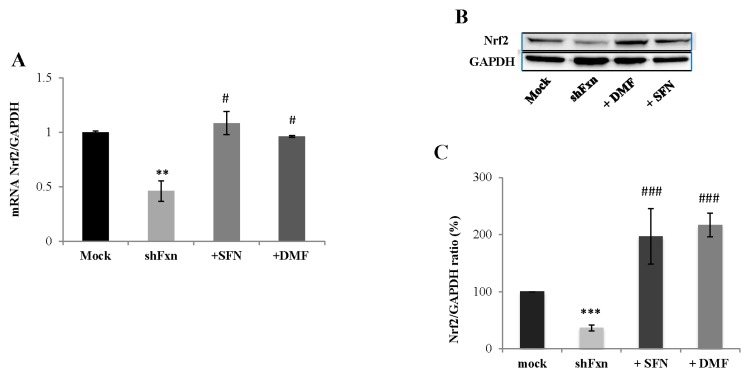
Nrf2-inducers up-regulate the Nrf2 expression in frataxin silenced neurons (shFxn). (**A**) qRT-PCR of mRNA Nrf2 levels in shFxn and Mock after 5 µM (24 h) sulforaphane (SFN) and 30 µM (24 h) dimethyl fumarate (DMF) treatments. Glyceraldehyde 3-phosphate dehydrogenase (GAPDH) is used for normalization and relative quantification of gene expression was performed according to the 2^−ΔΔ*C*t^ method; (**B**) Representative Western blot of DMF- and SFN-treated shFxn neurons; (**C**) Densitometry of Nrf2 protein amounts analyzed by Western Blot. Values represent the mean ± SD of three independent experiments (** *p* < 0.01 and *** *p* < 0.001, with respect to Mock; ^#^ *p* < 0.05 and ^###^ *p* < 0.001, with respect to untreated shFxn).

As shown in Figure 1, Nrf2 expression is down-regulated in shFxn neurons (54% decreased) and the treatment with 5 µM (24 h) SFN significantly increases its expression, either as mRNA (A, 2.3-fold) and as protein amount (B, C, 5-fold), with respect to untreated neurons. Also, the DMF, a well known inducer of Nrf2 already used in multiple sclerosis treatment and recently proposed as therapy in PD [20], led to 2- and 6-fold increases of Nrf2 mRNA (A) and protein expression (B, C) in 30 µM-treated shFxn (24 h).

### 2.2. SFN Activates the Phase II Response in shFxn

**Figure 2:**
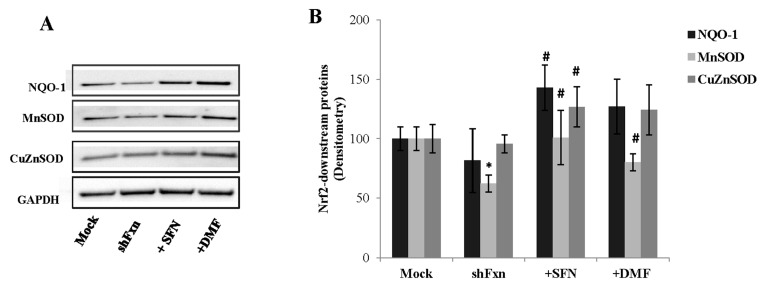
Nrf2 inducers activate the phase II response in shFxn. (**A**) Representative Western blot of downstream Nrf2 target proteins. An amount of 40 µg Mock, shFxn 5 µM (24 h) SFN and 30 µM (24 h) DMF treatments were applied onto 4–12% Bis–Tris SDS-polyacrylamide gel electrophoresis and probed with anti-NQO-1 (1:1000), MnSOD (1:5000) and Cu/ZnSOD (1:5000) antibodies; (**B**) Densitometry of blots, normalized to GAPDH. Values represent the mean ± SD of three independent experiments (* *p* < 0.05, with respect to Mock; ^#^ *p* < 0.05, with respect to untreated shFxn).

To fully assess the functional status of the Nrf2-ARE pathway, we analyzed the protein levels of three typical Nrf2-regulated phase II enzymes, NQO1, Cu/ZnSOD and MnSOD at 24 h SFN and DMF treatments (Figure 2). SFN was able to induce a consistent enhancement of their protein amounts (75% increase of NQO-1, 63% MnSOD, 34% Cu/Zn SOD, A, B). Comparable increases were elicited by the DMF treatment (56% enhancement of NQO-1, 29% MnSOD and 30% Cu/Zn SOD).

### 2.3. Nrf2-Inducers Target the Repletion of the Active Form of Glutathione (GSH)

**Figure 3:**
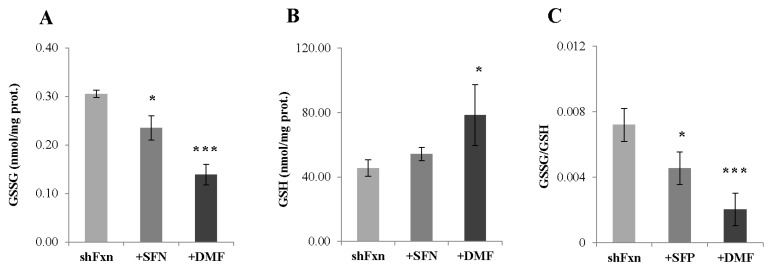
HPLC analysis of GSSG (**A**) and GSH (**B**) in shFxn after treatments with 5 µM (24 h) SFN and 30 µM (24 h) DMF. Nrf2 inducers cause repletion of the oxidized (GSSG)/reduced (GSH) glutathione ratio (**C**). * *p* < 0.05, *** *p* < 0.001, with respect to Mock.

As Nrf2 affects the glutathione synthesis by modulating the expression of the rate-limiting enzyme γ-glutamil cysteine ligase, and impaired glutathione homeostasis has been previously described in shFxn [13], we measured the GSSG/GSH ratio following SFN and DMF treatments (Figure 3). HPLC analysis evidenced a significant decrease of GSSG (22%) and a 20% increase of the reduced form (GSH) in SFN-treated shFxn. A consistent compensatory effect was also observed with DMF (55% GSSG decrease and 72% GSH increase), thus indicating that both drugs may restore the redox balance in frataxin-silenced neurons (as demonstrated by the reduction of the GSSG/GSH ratio, C).

### 2.4. SFN Triggers Axonal Re-Growth in the shFxn

**Figure 4:**
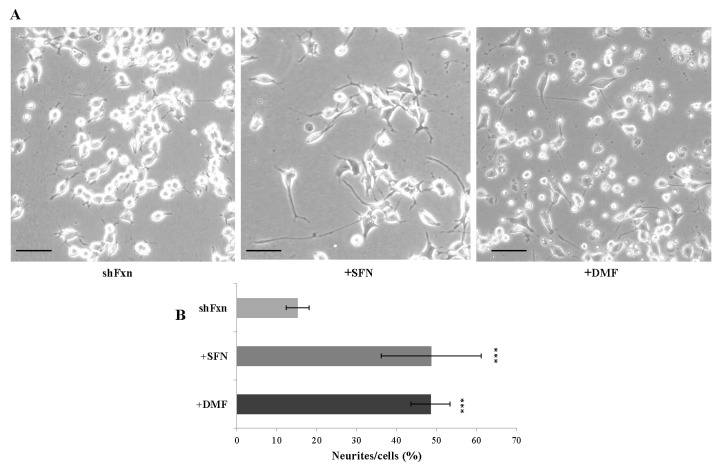
Nrf2-inducers trigger axonal re-growth in the shFxn. (**A**) Bright field photographs of untreated shFxn, 5 µM (24 h) SFN and 30 µM (24 h) DMF treated cells. A re-organization of the neurites network is evident; (**B**) The number of neurites has been measured by ImageJ software and normalized for number of cells (*** *p* <0.001; bar = 100 μm).

Frataxin silencing affects the neuronal morphology in shFxn, resembling the GSSG-induced axonopathy previously described in cultured motor neurons [14,21]. Thus, as glutathione balance was restored by Nrf2 activation, we wonder if Nrf2 inducers may also be effective on the axonal morphology. As reported in Figure 4, both SFN and DMF treatments trigger the axonal re-growth in shFxn neurons, with a mild re-organization of the network (A) and a consistent increase of neurites’ number (B).

### 2.5. SFN Increases the Frataxin Expression in shFxn

**Figure 5:**
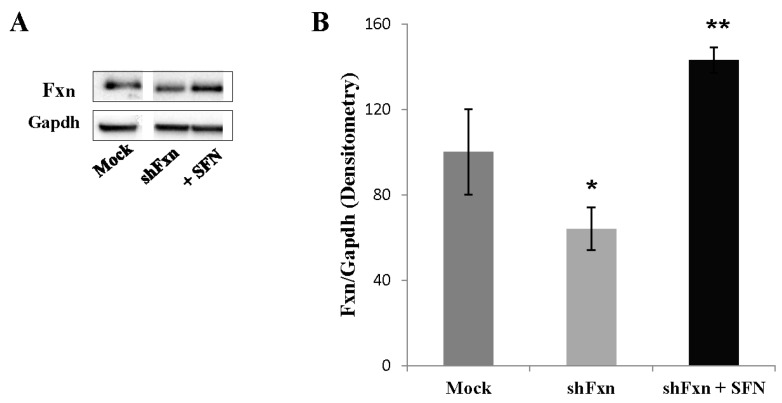
The amount of frataxin increases after SFN treatment. (**A**) Representative Western blot of shFxn incubated for 24 h with 5 µM SFN; (**B**) Densitometry of blots normalized to GAPDH. Values represent the mean ± SD of three independent experiments (* *p* < 0.05, ** *p* < 0.01, with respect to untreated shFxn).

Because the frataxin expression significantly correlated with Nrf2 expression in FA cell models [2,10,11], we next asked whether frataxin expression can be modulated by SFN. As showed in Figure 5, SFN induces a significant increase (2-fold) of the frataxin protein level in shFxn, when compared to untreated shFxn.

### 2.6. SFN Restores Nrf2 Transcriptional Activity in Fibroblasts of Patients with FA

**Figure 6:**
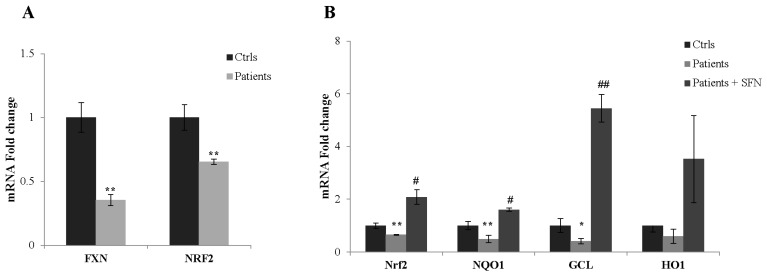
Expression of NRF2 and its down-stream genes in fibroblasts of patients with FA. Fibroblasts of three patients expressing a 35% residual amount of frataxin and 65% of NRF2 (**A**), with respect to healthy subjects, were treated with 10 µM SFN (24 h), and mRNA levels of NRF2-downstream-genes were determined (**B**). * *p* < 0.05 and ** *p* < 0.01, with respect to control fibroblasts; ^#^ *p* < 0.05 and ^##^ *p* < 0.01, with respect to untreated FA fibroblasts.

To test whether our findings are linked to human disease, also considering the decreased expression of Nrf2-targeted genes found in FA cultured fibroblasts [11], we then extended our studies to fibroblasts obtained from skin biopsies of three patients with FA (Figure 6A). The NRF2 expression was significantly increased in fibroblasts of patients after SFN treatment (1.5-fold, B). Also, the qRT-PCR analysis of NRF2-downstream genes showed consistent increases of transcripts (68% NQO-1, 90% HO-1) in SFN-treated fibroblasts, including the GCL mRNA (52% increase) which is critical for maintaining the glutathione homeostasis in cells (B).

### 2.7. NRF2 as a Potential Biomarker in FA

**Figure 7:**
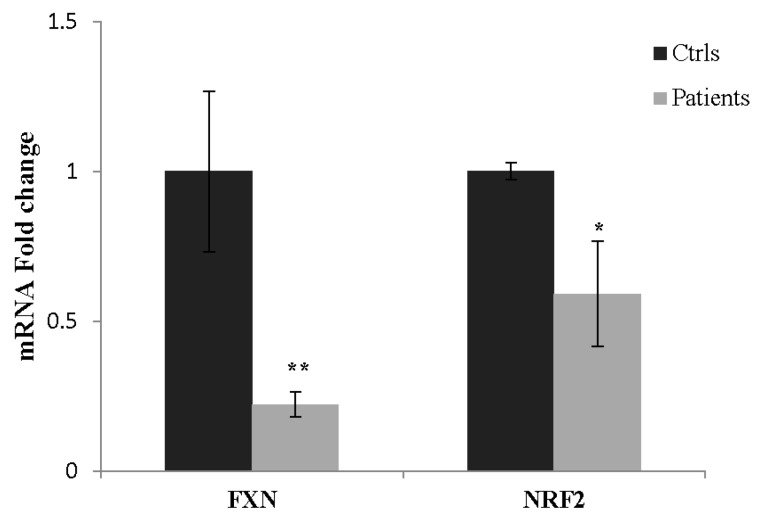
Frataxin and NRF2 mRNA levels in blood of patients with FA. qRT-PCR performed on PBMCs isolated from blood of FA patients (*n* = 4) evidenced 35% and 59% residual amounts of Fxn and NRF2 expression, respectively, compared with healthy subjects (*n* = 5) (* *p* < 0.05,** *p* < 0.01, respect to healthy subjects).

In order to evaluate whether the impairment of the Nrf2 pathway could be reflected at the systemic level, we analyzed the NRF2 expression in PBMCs of FA patients. As showed in Figure 7, the NRF2 expression was 41% reduced in blood of FA patients, thus evidencing that molecular events underlying pathological processes in FA are not locally restricted, but mirrored at the periphery. These findings lead us to propose Nrf2 as a blood redox sensor potentially useful in monitoring clinical trials.

## 3. Discussion

This study can be summarized as two main findings: (1) the Nrf2/ARE pathway is dysfunctional in shFxn neurons, creating a toxic oxidative stress environment; (2) SFN promotes activation of Nrf2/ARE signaling, reversing the oxidative stress environment and the neurodegenerative phenotype derived from frataxin deficiency.

Nrf2 is the guardian of redox homeostasis in cells and its regulation is a multifaceted pathway that ultimately provides a tightly controlled protective mechanism. Upon exposure to stressor molecules, such as electrophiles or free radicals, Nrf2 is released from Keap1 and triggers the signaling for the gene induction. Like many other transcription factors, Nrf2 contains cysteine residues that are redox sensitive (through their thiol groups) and are essential for the nuclear translocation and the recognition of their DNA binding domain [22]. Cysteine residues reactive to changes in the GSH/GSSG redox couple have been identified in the purified human Keap1 protein and defined as “sensors” of oxidative stress [23]. The oxidation of these cysteines results in the inhibition of the Nrf2 nuclear re-distribution and/or DNA binding, thus a balanced redox environment is required to avoid the oxidation of these reactive cysteines and to guarantee the Nrf2 functionality. The presence of nuclear TRX and Ref-1 promotes these favorable redox conditions [24]. Additionally, Nrf2 undergoes auto-regulation through post translational modifications, thereby greatly amplifying its antioxidant response [25,26].

The redox imbalance is a common condition of several SNC pathologies and maintaining cellular redox homeostasis is critical for the prevention of neurodegeneration. For this reason, neurodegenerative diseases represent excellent candidates for Nrf2-targeted treatments and a wide range of compounds are able to induce Nrf2 activation [27,28,29]. Nrf2 inducers have been found to be neuroprotective in the MPTP model of PD [18,30,31], in fragile X syndrome [32], in multiple sclerosis [33,34], and a clinical trial with a novel Nrf2 activator (RTA 408) has been started for the treatment of FA (REATA Pharmaceuticals, ClinicalTrials.gov, NCT02255435). Protective effects mediated by Nrf2 induction have been evidenced also in Alzheimer’s disease models after SFN treatment, or in Huntington’s disease, where the Nrf2 activation contributes to improve mitochondrial function. The Nrf2 pathway plays a pivotal role also in the neurodegenerative processes of Amyotrophic Lateral Sclerosis and its systemic activation delayed disease progression in a transgenic mouse model of the disease [35]. A potential positive effect of Nrf2 inducers has even been reported in a murine model of Spinal Muscular Atrophy, in which a mild improvement of neuromuscular phenotype and survival was observed [36].

In FA, the dys-regulation of cellular antioxidant defenses, due to frataxin deficiency, exacerbates oxidative stress, thus the Nrf2 activation becomes more and more of an attractive strategy for the treatment of this disease.

Previously, we identified antioxidant defects in a neuronal model of FA obtained by frataxin silencing mouse motor neurons [10,13,14]. Here, we used this model to investigate the potential therapeutic benefit of SFN, an up-regulator of the transcriptional activity of antioxidant genes also operating in restoration of the active chromatin structure [37]. We analyzed the SFN-driven induction of Nrf2 by estimating the expression of the antioxidant enzymes NQO1, a key player in the detoxification of reactive quinones, and SOD [1/2], both of them significantly reduced in the FA neuronal model [38,39]. We found that 24 h treatment with SFN increased transcript and protein levels of the enzymes, thus strongly supporting the efficacy of this molecule in reactivating the Nrf2 signaling both at transcriptional and translational levels.

SFN, an isothiocyanate obtained from cruciferous vegetables, is one of the most potent naturally occurring Nrf2 activators. It is neuroprotective against nigral dopaminergic cell death, astrogliosis, and microgliosis in the MPTP mouse model of PD [15,18]. A SFN-driven neuroprotective effect was also reported in a 6-OHDA-induced acute mouse model, mainly attributed to its ability to enhance glutathione levels and glutathione-dependent enzymes [40]. In our model, the treatment with SFN has been shown to re-equilibrate the glutathione homeostasis by consistently decreasing GSSG levels. In light of our findings, we expect that balancing redox conditions in cells may re-activate overall the antioxidant machinery in FA, ultimately restoring neuronal health and functionality.

However, the Nrf2 signaling cascade is complex [26] and simultaneous mechanisms may be involved in the Nrf2-induction observed in FA: the classical Nrf2-Keap1 signaling pathway, by direct modifications of critical Keap1 cysteines [41,42,43]; the post-translational regulation of Nrf2 through phosphorylation [44] and/or ubiquitination [45]; the transcriptional modulation of the Myc and Jun binding to the Nrf2 promoter [46]; the ribosomal internalization of Nrf2 mRNA [47], and the demethylation of promoter CpGs [48].

On the other hand, a broad spectrum of actions has been shown for SFN, including an emerging mechanism that involves the epigenetic control of histone modification and DNA methylation [37,49]. Additionally, SFN has been found to inhibit protein tyrosine phosphatases (PTPs), thus playing a central role also in protein phosphorylation [50,51], a mechanism engaged in frataxin degradation [52]. Hence, multiple mechanisms may be simultaneously activated by SFN and further investigations are needed to elucidate its protective role in FA.

Overall, our findings support Nrf2 as a therapeutic target for FA, and its induction as a promising approach to prevent or slow the pathological changes observed in this disease. Furthermore, the Nrf2 impairment mirrored at the systemic level in PBMCs of patients may help to open new perspectives for biomarker research in FA, potentially useful for monitoring clinical trials.

## 4. Materials and Methods

SFN (LKT Laboratories, St. Paul, MN, USA) and DMF (Sigma-Aldrich, St. Louis, MO, USA) were prepared in DMSO. Lentiviral particles encoding short-hairpin RNA (shRNA) sequences have been purchased from Open Biosystem (Waltham, MA, USA).

### 4.1. Stable shFxn Cell Lines Generation and Treatments

Frataxin silencing and stable shFxn cell lines generation have been performed as described in Piermarini et al. [14]. Briefly, following the methodology used in Carletti et al. [13], we silenced the mouse NSC34 motor neurons for the frataxin gene with shFxn lentiviral vectors generating a cell line (called shFxn) displaying 40% residual frataxin levels. Cell sorting for GFP expression has been performed 96 h after infection with shRNA lentiviral particles by using a FACSAria II cell sorter (BD Biosciences, Franklin Lakes, NJ, USA). Positive cells have been collected and exposed to puromycin selection (10 µg/mL), which was maintained for 7 days to obtain a stable cell line that has been finally analyzed for frataxin silencing. Control cell lines (referred to as Mock) consist of cells infected with the GFP vector alone. For treatments, neurons were differentiated as in Carletti et al. [21], incubated for 24 h with 5 µM SFN or 30 µM DMF diluted in culture medium, pelleted by centrifugation and subjected to electrophoresis and HPLC analysis, as reported below.

### 4.2. Fibroblasts Cultures

Skin biopsies were taken from three clinically affected (and genetically proven) FRDA patients (two males and one female) (Table 1) and three age-matched controls. Fibroblasts were grown in Dulbecco’s modified Eagle’s medium supplemented with 10% fetal bovine serum, 50 units/mL penicillin, 50 µg/mL streptomycin, 0.4% (*v*/*v*), at 37 °C in 5% CO_2_. Fibroblasts were cultured to 70% confluence as reported in [50] and incubated for 24 h with 10 µM SFN diluted in culture medium. After washing, cells were lysated in 1 mL TRI Reagent (Sigma-Aldrich, St. Louis, MO, USA) for RNA extraction and subjected to quantitative Real-Time PCR. The assays were performed in triplicate and cells were used at similar passage numbers.

### 4.3. Blood Sample Collection

Blood samples from 4 patients (Table 1) were collected into EDTA Vacutainer Tubes (Becton Dickinson, Rutherford, NY, USA) and PBMC was isolated by adding 10% dextran. After 45 min at room temperature, the upper phase was centrifuged at 2600× *g* (5 min) and the pellet washed with 0.9% NaCl and stored at −20 °C until the analysis. All the participants signed an informed consent and the study was approved by the Ethics Committee of “Bambino Gesù” Children’s Hospital (code 1166/2016; date of approval 08/06/2016).

### 4.4. Quantitative Real-Time PCR (qRT-PCR)

Total RNA was extracted from shFxn, fibroblasts and PBMC using TRI Reagent (Sigma-Aldrich, St. Louis, MO, USA), according to the manufacturer’s protocol. An amount of 1 µg of each RNA samples was reverse transcribed with the SuperScript™ First-Strand Synthesis system and random hexamers as primers (Life Technologies, Carlsbad, CA, USA). The expression levels of FXN, NRF2, NQO1, HO-1, GCL were measured by qRT-PCR in an ABI PRISM 7500 Sequence Detection System (Life Technologies, Carlsbad, CA, USA) using Power SYBR Green I dye chemistry. Data were analyzed using the 2^−ΔΔ*C*t^ method with TBP (TATA box binding protein) and GAPDH as housekeeping genes, and data are shown as fold change relative to controls. Primers used for qRT-PCR are reported in Table 2.

### 4.5. Immunoblot Analysis

Cells were homogenized and lysed on ice with RIPA buffer, including DTT and protease inhibitors. For Western blotting analysis, 40 μg of proteins was subjected to SDS PAGE on 4–12% denaturing gel and probed with the following antibodies: NRF2 (1:500, Abcam, Cambridge, UK), NQO1 (1:1000, Novus Biologicals, Minneapolis, MN, USA), SOD 1/2 (1:5000, Stressgen, Victoria, BC, Canada), Frataxin (1:500, Santa Cruz Biotechnology, Dallas, TX, USA) and GAPDH (1:15,000, Sigma Aldrich, St. Louis, MO, USA) as loading control. Immunoreactive bands were detected using the Lite Ablot Extend Long Lasting Chemiluminescent substrate (Euroclone, MI, Italy). Signals derived from appropriate HRP-conjugated secondary antibodies (Bethyl Laboratories, Montgomery, TX, USA) were captured by Chemi DocTM XRS 2015 (Bio-Rad Laboratories, Hercules, CA, USA) and densitometric analysis was performed using Image Lab software (Version 5.2.1; © Bio-Rad Laboratories).

### 4.6. HPLC Analysis of Reduced (GSH) and Oxidized (GSSG) Glutathione Forms

GSH and GSSG levels were measured as previously reported [53]. In brief, cells were sonicated and de-proteinized by adding 12% sulfosalicylic acid, and the acid-soluble fraction was used for GSH determination. For GSSG measurements, cell sonication was performed in buffer containing 10 mM *N*-ethylmaleimide. Derivatization and chromatography procedures were described in Pastore et al. [54].

### 4.7. Neurite Numbers’ and Morphology

For the experiments, cells were cultured at a density of 2000 cell/cm^2^ on poly-d-lysine (100 µg/mL) pre-coated tissue culture glass cover slips and differentiated as reported [21]. To measure neurites’ number, shFxn neurons (7 days differentiation) were incubated for 24 h with 5 μM SFN and 30 μM DMF, and micrographs were acquired by Olympus IX 70 microscope (software I.A.S. 2000). Quantitative evaluations were made using the ImageJ software (1.47 v, Bethesda, MD, USA). We examined neurites of shFxn cultures (507 cells), SFN and DMF treatments (400 and 350 cells, respectively) from four independent experiments.

### 4.8. Statistical Analysis

Statistical analysis was performed using the GRAPHPAD/Prism 5.0 Software (San Diego, CA, USA). Statistically significant differences between groups were analyzed using Student’s *t*-test for normally distributed variables. All data are presented as mean ± standard deviation. Statistical significance was defined as * *p* < 0.05, ** *p* <0.01, *** *p* < 0.001, compared to healthy controls, and ^#^ *p* < 0.05, ^##^ *p* <0.01, ^###^ *p* < 0.001, compared to untreated cells.

## Figures and Tables

**Table 1:** Clinical data of patients with FA.

Case	Age	Sex	Genetics	Onset	Duration	SARA	8 m	9HPTdom	PATA	Weight	Height
FV	14	F	780/1114	5	9	12.5	6.9	48.0	--	28	155
BD	9	F	1116/1116	1	8	10	6.8	44.0	17	30	130
MA	15	M	999/766	8	7	12	9.3	47.7	24	63	159
DG	32	M	c.410G>T(p.Gly137Val)4/281	23	9	8.5	5.3	36.3	33	70	174

**Table 2:** Primers used for qRT-PCR.

**Mouse Genes**	**Sequence (5′→3′)**	
*FXN*	Fw-CCTGGCCGAGTTCTTTGAAG	Rv-GCCAGATTTGCTTGTTTGG
*NRF2*	Fw-TGGAGGCAGCCATGACTGA	Rv-CTGCTTGTTTTCGGTATTAAGACACT
*TBP*	Fw-CTCTGACCACTGCACCGTT	Rv-CTGCAGCAAATCGCTTGGG
*GAPDH*	Fw-CCTCGTCCCGTAGACAAAATG	Rv-TGAAGGGGTCGTTGATGGC
**Human Genes**	**Sequence (5′→3′)**	
*FXN*	Fw-CCTTGCAGACAAGCCATACA	Rv-CCACTGGATGGAGAAGATAG
*Nrf2*	Fw-ACACGGTCCACAGCTCATC	Rv-TGTCAATCAAATCCATGTCCTG
*GCL*	Fw-TTGCCTCCTGCTGTGTGATG	Rv-ATCATTGTGAGTCAACAGCTGTATGTC
*HO-1*	Fw-CTCAACATCCAGCTCTTTGAG	Rv-AATCTTGCACTTTGTTGCTGGC
*NQO1*	Fw-GACATCACAGGTAAACTGAAGG	Rv-GCAGGGGGAACTGGAATATC
*TBP*	Fw-CCGAAACGCCGAATATAATCC	Rv-AAATCAGTGCCGTGGTTCGT
*GAPDH*	Fw-GATGACATCAAGAAGGTGGTG	Rv-GTCATACCAGGAAATGAGCTTG
